# Kartogenin regulates hair growth and hair cycling transition

**DOI:** 10.7150/ijms.68434

**Published:** 2022-03-06

**Authors:** Yuhong Chen, Lijuan zhou, Yuxin Ding, Xiaoshuang Yang, Jing Jing, Xianjie Wu, Jufang Zhang, Zhongfa Lu

**Affiliations:** 1 Department of Dermatology, The Second Affiliated Hospital, School of Medicine, Zhejiang University, Hangzhou 310009, PR China.; 2 Department of Dermatology, Huashan Hospital, Fudan University, 12 Middle Wulumuqi Road, Shanghai 200040, PR China.; 3 Department of cosmetic & plastic surgery, Affiliated Hangzhou First People's Hospital, Zhejiang University School of Medicine, Add: 261 Huansha road, Hangzhou 311100, PR China.

**Keywords:** Kartogenin, Hair growth, Hair follicle, Outer root sheath cell, Transforming growth factor-β signaling

## Abstract

**Background:** Kartogenin is a heterocyclic compound able to promote the proliferation, migration, and differentiation of various cell types and induce cartilage-like tissue regeneration. However, the role of kartogenin in hair follicles (HFs), remains unknown. We therefore investigated the effects of kartogenin on the regulation of hair growth and hair growth cycle transition.

**Methods:** The effects of kartogenin on the proliferation, cell cycle status, and migration of primary human outer root sheath cells (ORSCs) were evaluated by MTS assay, flow cytometry, Transwell^®^ and scratch assays, respectively. We exposed ORSCs to kartogenin (1 µM) and determined changes in mRNA and protein levels of transforming growth factor (TGF)-β2/Smad signaling molecules by reverse transcription polymerase chain reaction, western blotting, and immunofluorescence. We also examined the effects of kartogenin (10 µM) on HFs in mice by histology following cutaneous injection.

**Results:** Kartogenin enhanced ORSC proliferation and migration function in a dose-dependent manner, and downregulated the expression of TGF-β2/Smad signaling molecules *in vitro*. Injection of kartogenin delayed catagen phase and increased regenerated hair length in mice *in vivo*.

**Conclusions:** Kartogenin modulates HF growth and regulates the hair cycle and the TGF-β2/Smad signaling pathway, providing a potential new approach for the treatment of hair loss.

## Introduction

The hair growth cycle includes three phases: a growth phase (anagen phase), regression phase (catagen phase), and quiescence phase (telogen phase) 1. During anagen phase, epithelial proliferation occurs together with thickening, elongation, and pigmentation of the hair shaft, while catagen phase is characterized by rapid apoptosis, and telogen is a relatively quiescent phase 2. Hair loss (alopecia) affects the sufferer's appearance and can have serious effects on their mental health 3. Androgenic alopecia (AGA) is the most common type of hair loss, characterized by a shorter anagen phase and miniaturization of hair follicles (HFs) 1,4. Current therapies for alopecia are thus mainly based on extending the anagen phase and enlarging the hair bulb 5. However, existing drug therapies have some limitations 6. Finasteride is only useful for alopecia caused by abnormal androgens (increased conversion from testosterone to dihydrotestosterone), and may have side effects associated with androgen blockage, while its usen in women is still limited 7,8. Although minoxidil acts as a widely used clinical drug for AGA, the effects are not permanent and require long term topical use of the medication 9. There is thus an urgent need to find more effective and safe methods for treating hair loss.

Kartogenin is a recently synthesized small heterocyclic compound 10,11 shown to promote the proliferation, migration, and differentiation of various cell types 12 such as chondrocyte, mesenchymal stem cells, and to promote cartilage-like tissue regeneration 13,14. Decker et al. demonstrated that kartogenin increased chondrocyte proliferation, migration, and differentiation via activation of the hedgehog and transforming growth factor (TGF)-β signaling pathways 15. However, to the best of our knowledge, the function of kartogenin in regulating hair growth and the HF cycle remains unclear.

Outer root sheath cells (ORSCs) act as a reservoir for various epithelial stem cell populations and play a critical role in the maintenance and development of the HF. ORSCs are regarded as precursor cells of interfollicular epidermal keratinocytes 16 and play a critical role in skin stem cell biology and matrix replenishment 17,18.

The functions of ORSCs are regulated by various signaling molecules, among which the transforming growth factor (TGF)-β2 is crucial 19-21. TGF-β2, which is a multifunctional cytokine, propagates signals via TGF-β receptor and phosphorylating Smad2 and Smad3 22. Various studies demonstrated that TGF-β2 is a catagen phase inducer and inhibition of TGF-β2 activity during hair cycle could suppress the transition from anagen phase to catagen phase and prolong anagen phase 19.

In this study, we investigated the function of kartogenin in hair growth and HF cycle regulation. The results increased our understanding of the role of kartogenin in the regulation of hair growth and the HF cycle, and may provide a potential new approach for the treatment of hair loss.

## Materials and methods

All protocols for handling of human tissue were approved by the Zhejiang University School of Medicine Second Affiliated Hospital Institutional Review Board.

### Isolation and culture of normal ORSCs from human scalp

We followed the methods of Lijuan Zhou et al. 2018 23,24. Scalp specimens were obtained from healthy human (n = 15; 10 females and 5 males; age range, 20-50 years) who were undergoing cosmetic surgery, with informed consent. ORSCs were isolated from the scalp specimens by two-step enzyme digestion. ORSCs were centrifugation at 800 × *g* for 10 min and then resuspended in defined keratinocyte serum-free medium supplemented with keratinocyte growth factor (Gibco, Invitrogen, USA). ORSCs were cultured at 37 °C and 5% CO_2_. The medium was changed twice a day. Cells were passaged at 70-80% confluence and were used in passage four for the following experiments.

### Cell proliferation assay

The cells were seeded into 96-well plates at the concentration of 1.5 × 10^4^/well. After cultured with SFM supplemented with kartogenin at 0, 0.5, or 1 µM for 24h, 20 μl 3-(4,5-dimethylthiazol-2-yl)-5-(3-carboxymethoxyphenyl)-2-(4-sulfophenyl)-2H-tetrazolium (MTS) (Promega) was added to each well at the last 30 min of incubation. The wavelength of 490 nm was used to measure light absorption value of each well by ELX808 microplate reader (BioTek, Winooski, VT, USA).

### Cell cycle analysis by flow cytometry

After kartogenin treatment (0 or 1 µM) for 24 h, cells were re-suspended in PBS and were subsequently fixed in 75% pre-cooled ethyl alcohol for 8h at 4 °C. Cells were then centrifuged for 5 min at 1000 rpm, rinsed trice with PBS, dyed with propidium iodide-RNase using a commercial kit (BD Biosciences, San Jose, CA, USA), according to the manufacturer's protocol. The stained cells were analyzed by BD FACSCaliburTM flow cytometer (BD Biosciences, San Jose, CA) and data was analyzed using CytExpert software. Data were presented by percentage of cells compared to the control group.

### Transwell^®^ migration assay

200 uL ORSCs cells (1 × 10^6^/ mL) were suspended in basic keratinocyte medium without keratinocyte growth factor and plated into the upper chamber. The lower chamber was added with 500uL SFM containing 0 or 1 µM kartogenin. Following 24 h of incubation at 37 °C, the invasive cells were fixed with 4% paraformaldehyde for 20 min at room temperature and stained with crystal violet for 5 min. Stained cells were visualized and counted using an inverted microscope (Olympus, Tokyo, Japan) in five randomly selected fields of view.

### Wound healing assay

ORSCs (2 × 10^5^ cells/well) were plated into a 6‑well plate and cultured until they reached 100% confluency. A 200‑µl sterile pipette tip was subsequently used to make a vertical scratch. The cells were then washed with PBS three times to remove non‑adherent cells and incubated with SFM supplemented with or without (0 or 1 µM) for 24 h. The migration distances were captured after 0, 12, and kartogenin 24 h using a microscope (Olympus, Tokyo, Japan) and assessed using Adobe Photoshop CS3 software (Adobe Inc., San Jose, CA, USA).

### Reverse transcription-polymerase chain reaction (RT-PCR)

After kartogenin treatment (0 or 1 µM), total RNA was extracted using TRIzol reagent (Ambion, New York, NY, USA). Reverse transcription and RT-PCR was performed as described previously 23,24. The sequences of the specific primers (Sangon Biotech, Shanghai, China) used for RT-PCR are listed in Table 1. The mRNA expression levels of target genes were described relative to that of GAPDH using the 2^-ΔΔCt^ method.

### Western blot analysis

The protocol for western blot has been described previously 23,24. Primary antibodies were used as follows: rabbit polyclonal anti-TGF-β2 (1:250; Santa Cruz Biotechnology, Santa Cruz, CA, USA), and rabbit polyclonal anti-phosphorylated Smad2 (p-Smad2)/phosphorylated Smad3 (p-Smad3) (1:1000; Cell Signaling Technology, Danvers, MA, USA). GAPDH detected with rabbit monoclonal anti-GAPDH antibody (1:1000; Cell Signaling Technology, MA, USA) served as the loading control.

### Immunofluorescence

Cells were fixed with 4% paraformaldehyde for 15 min and washed three times with cold PBS, then blocked with 10% fetal bovine serum for 1 h at room temperature. 0.1% Triton X-100 was used for blocking for 10 min. ORSCs were then incubated with TGF-β2 (Abcam, Cambridge, MA, USA; 1:200), p-Smad2 and p-Smad3 (Cell Signaling Technology; 1:200) antibodies overnight (16h) at 4 °C. After incubation with fluorescence-conjugated secondary antibody (Jackson Laboratories; 1:200) for 2 h in the dark, nuclei were stained with DAPI (1:5000; Roche, China) for 5 min. Protein localization and expression in ORSCs were compared by fluorescence microscopy (EU5888; Leica, Wetzlar, Germany) and four randomly divided regions were required for quantitative analysis using ImageJ software. All immunofluorescence assays were repeated at least three times.

### Kartogenin injection in mice

We followed the methods of Lijuan Zhou et al. 2018 23,24 to induce anagen phase in female C57BL/6 mice. The mice were then randomized into vehicle (DMSO)- and kartogenin-treated groups (n = 6 each). To examine the anagen-to-catagen transition, mice were treated with 100 μL kartogenin (10 µM) dissolved in DMSO or vehicle (DMSO) once daily from p.d. 12-17 (total 600 μL) and sacrificed at p.d. 18 and p.d.21. Skin samples were collected for analysis and regrown hairs were plucked from depilated dorsal region at p.d.18 and p.d.21. We measured the average hair length from 30 hairs per mouse.

### Quantitative histomorphometry

Dorsal skin samples were fixed with formaldehyde solution and sectioned into 5-7 μm sections, which were stained with hematoxylin and eosin (HE), followed by analysis of hair length, hair bulb diameter, hair cycling score (HCS), and skin thickness using a light microscope. Based on previous studies 25, we assigned scores of 100, 200, and 300 for anagen VI, catagen II-III, and catagen IV-V phases HFs during anagen-to-catagen transition, respectively 26. At least 50 HFs per sample were analyzed.

### Statistical analysis

All experiments were repeated at least three times. Statistical analysis was performed using SPSS software (ver. 17.0; SPSS Inc., Chicago, IL, USA). Results were compared using Student's *t*-tests and one-way ANOVA. Results are presented as mean ± standard deviation (SD).

## Results

### Kartogenin stimulated proliferation and increased S, G2, and S/G1 phases in ORSCs *in vitro*

**Figure 1:**
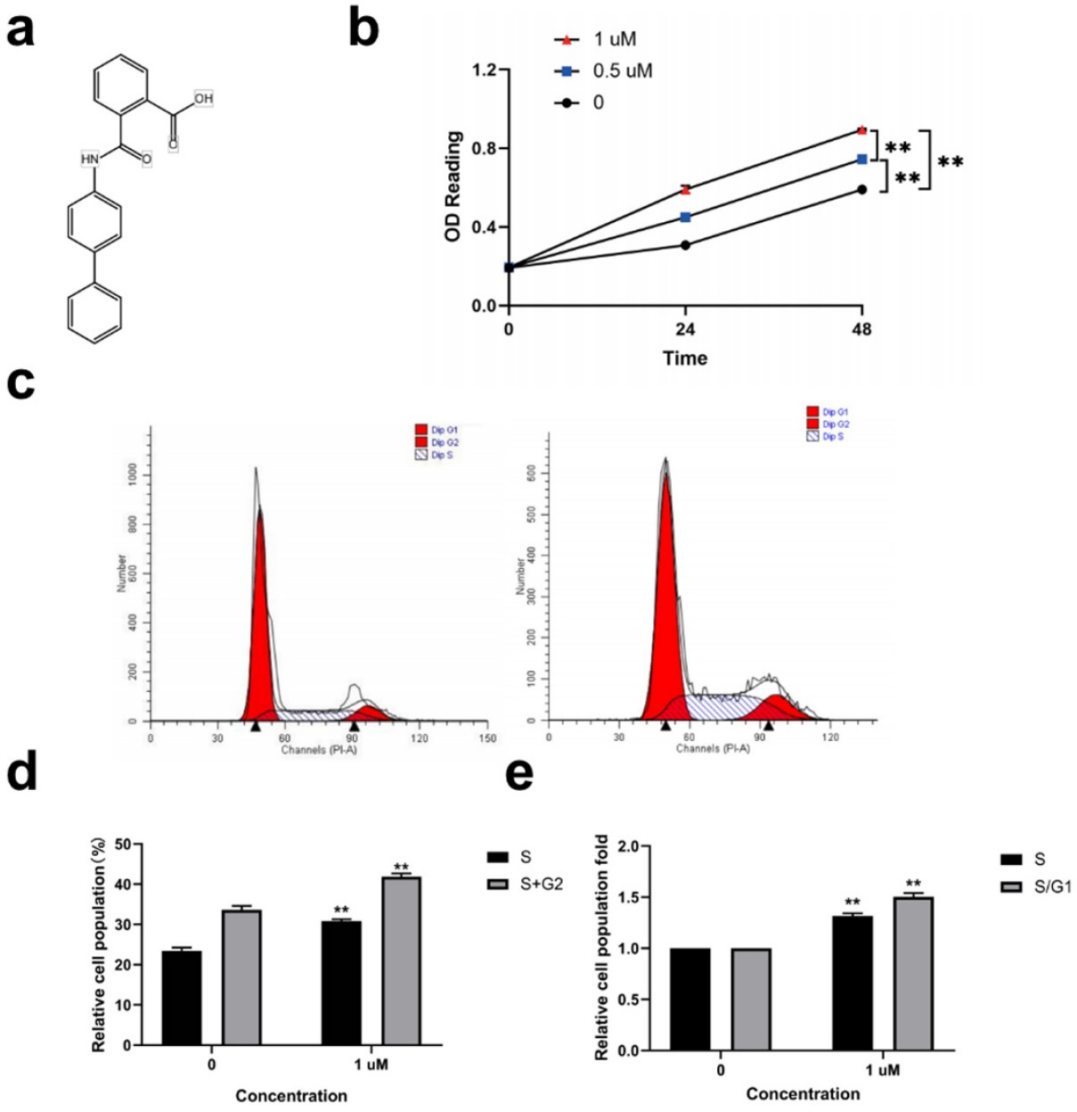
** Kartogenin treatment stimulated human ORSC proliferation and increased cells in S and S/G1 phases *in vitro*. a.** Structure of kartogenin. **b.** Kartogenin increased proliferation of ORSCs in a dosedependent manner, as measured by MTS assay. **c, d.** Distributions of ORSCs in G1, S, and G2 phases in the presence or absence of kartogenin, detected by flow cytometry. **e.** Fold changes in the fractions of cells in S and S/G1 phases with or without kartogenin treatment. Bars represent mean ± SD. **P < 0.01.

Kartogenin (Fig. 1a) (0.5, and 1 µM) promoted ORSC proliferation in a concentration-dependent manner compared with untreated control cells (Fig. 1b) (P<0.01). We then analyzed the effect of 1 µM kartogenin on the cell cycle status in ORSCs by propidium iodide-RNase staining and flow cytometry. The results showed that 41.88% of ORSCs entered the S and G2 phases following treatment with 1 µM kartogenin, compared with only 33.64% of vehicle-treated cells (P<0.01) (Fig. 1c, d). Treatment with kartogenin 1 µM resulted in 1.32-fold (P<0.01) increases in the S-phase fraction and 1.50-fold (P<0.01) increases in the S/G1 fraction, respectively (Fig. 1e). These results demonstrated that kartogenin stimulated ORSC proliferation.

### Kartogenin stimulated ORSC migration function *in vitro*

**Figure 2:**
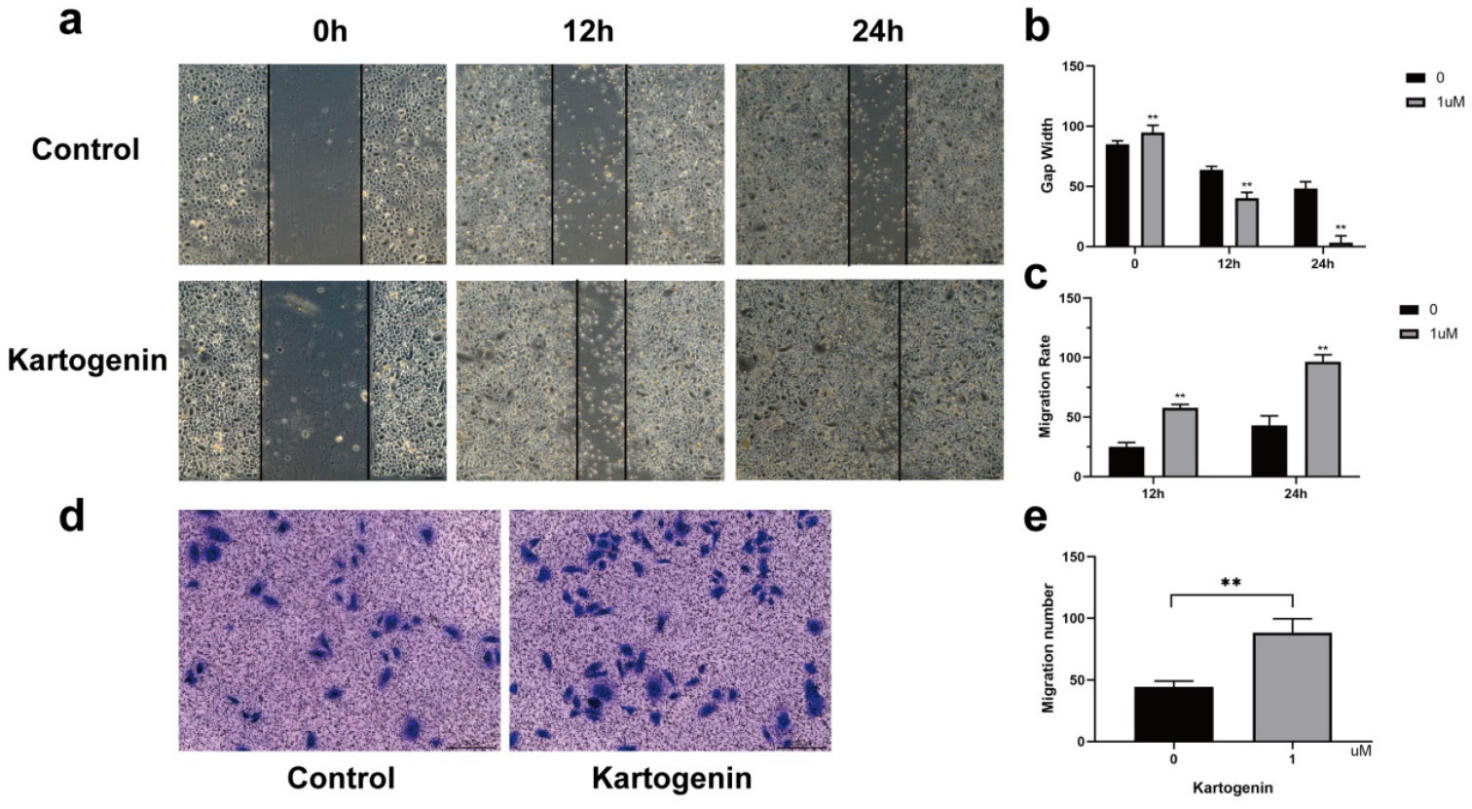
** Kartogenin increased human ORSC migration function. a-c.** Wound scratch assays of ORSCs cultured with or without kartogenin treatment. **d-e.** Transwell assays of ORSCs with or without kartogenin treatment. Data represent mean ± SD. **P < 0.01.

Kartogenin has been shown to coordinate cellular migration and proliferation during cartilage regeneration 12,15. We therefore determined if kartogenin stimulation increased ORSC migration and wound closure using *in vitro* Transwell^®^ and scratch assays, respectively. Kartogenin 1 µM promoted ORSC migration relative to untreated control cells (P<0.01), as demonstrated by wound healing assays (Fig. 2a-c) and Transwell^®^ (Fig. 2d-e).

### Kartogenin downregulated expression of TGF-β2/Smad signaling molecules in ORSCs *in vitro*

**Figure 3:**
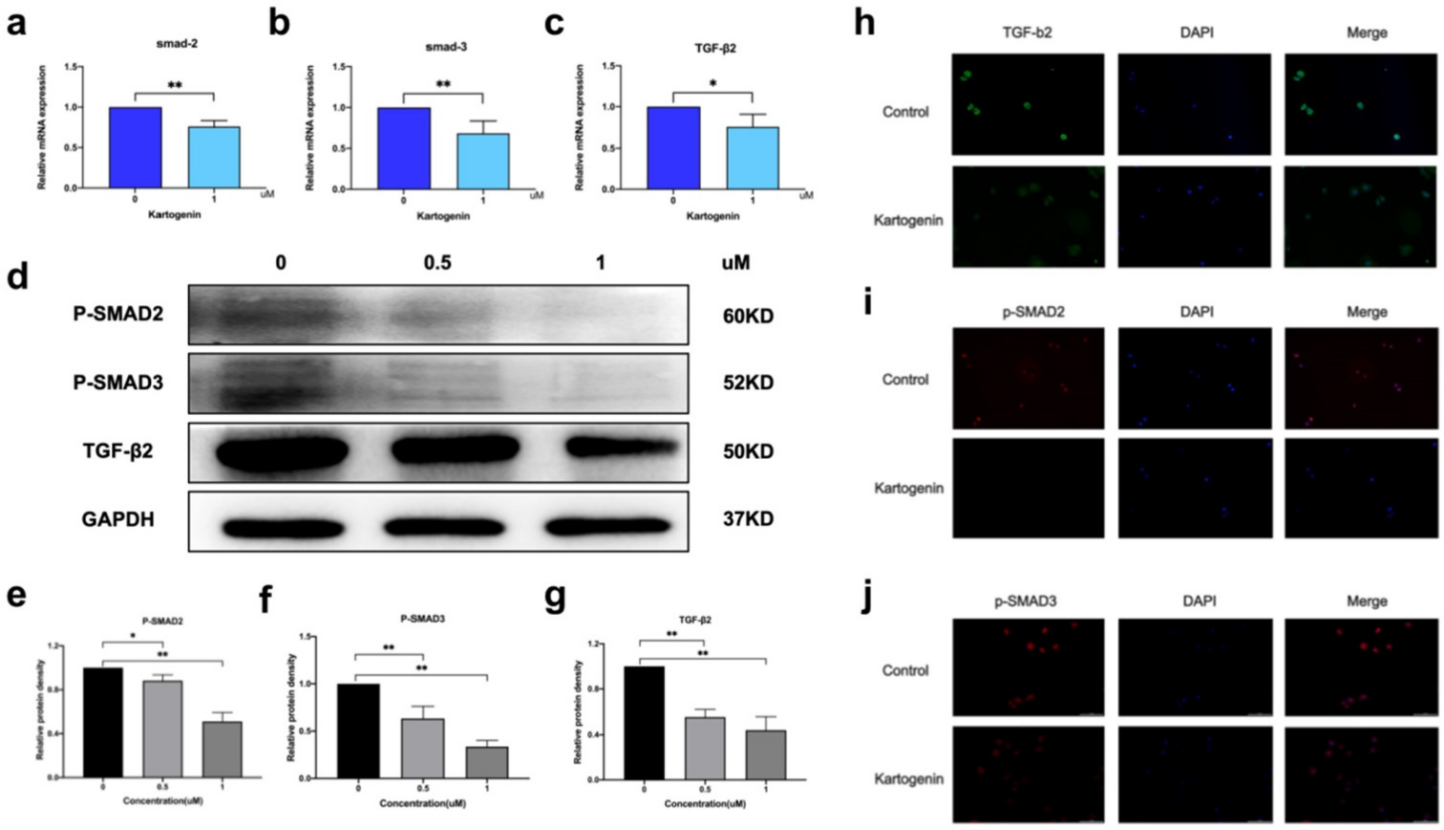
** Kartogenin reduced the mRNA and protein levels of TGF-β2/Smad signaling molecules. a-c.** Relative mRNA expression levels of TGF-β2, Smad2, and Smad3 in human ORSCs, determined by RT-PCR. **d.** TGF-β2, p-Smad,2 and p-Smad3 protein expression levels in human ORSCs, detected by western blotting. The grouping of blots was cropped from different gels. **e-g.** Quantitative analysis of TGF-β2, p-Smad2, and p-Smad3 protein levels. **h-j** TGF-β2 shown in green, p-Smad2, and p-Smad3 shown in red and nuclei counterstained with DAPI (blue). Merged images indicate the expression and location of TGF-β2, p-Smad2, and p-Smad3.*P < 0.05, **P < 0.01.

P-Smad2/p-Smad3 form a heteromeric complex with Smad4, which then translocate to the nucleus and regulates the expression of several genes 7. We examined the mRNA and protein levels of TGF-β2/Smad signaling molecules in ORSCs treated with kartogenin for 24 h, using RT-PCR and western blotting, respectively. Kartogenin treatment decreased TGF-β2, p-Smad2, and p-Smad3 protein expression levels in dose-dependent manners (Fig. 3d, Sup Fig. 1). At a concentration of 1 uM, the protein level of TGF-β2, p-Smad2, and p-Smad3 were decreased 0.66 -, 0.49-, 0.67-fold (P<0.01), respectively, compared with control cells (Fig. 3e-g). Similar trends were observed in mRNA levels of TGF-β2, Smad2, and Smad3, which were decreased 0.75-fold (P<0.05), 0.76-fold (P<0.01), and 0.68-fold (P<0.01), respectively (Fig. 3a-c). The localization of TGF-β2 in ORSCs after treatment with 0 or 1 µM kartogenin for 24 h was determined by cellular fluorescence. TGF-β2 was expressed in both the nuclei and cytoplasm of untreated control ORSCs, most notably in the cytoplasm (Fig. 3h), while p-Smad2 and p-Samd3 also showed strong nuclei signals (Fig. 3i-j). In contrast, TGF-β2, p-Smad2, and p-Smad3 expression levels were all reduced after treatment with kartogenin (Fig. 3e-g). The results of quantitative analyses of TGF-β2, p-Smad2, and p-Smad3 were consistent with the above results.

Taken together, these results thus demonstrated that kartogenin downregulated the TGF-β2/Smad signaling pathway.

### Local injection of kartogenin into HFs in anagen phase delayed entry into catagen phase *in vivo*

**Figure 4:**
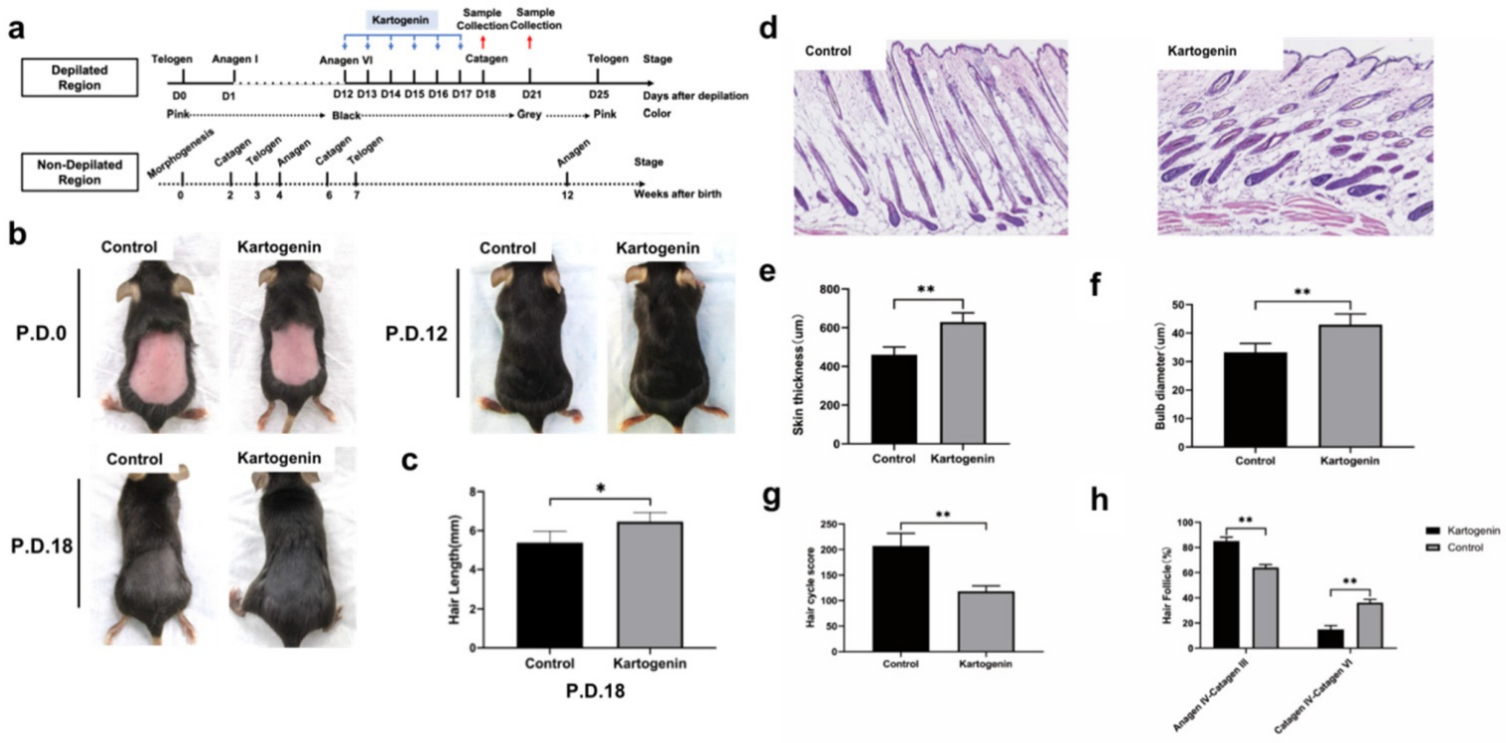
** Injection of kartogenin delayed transition of mouse HFs from anagen phase to catagen phase.** Kartogenin (0, 1 µM in 100 µL) was injected subcutaneously into mouse dorsal skin at p.d. 12 (100 µL over 6 days, total 600 µL). Results represent mean ± SD (n = 6 per group). *P < 0.05, **P < 0.01. **a.** Experimental time course. **b.** Macrophotograph of control group and kartogenin group at p.d.0, p.d.12 and p.d.18. **c.** Hair length in dorsal skin. d. H&E staining images of control group and kartogenin group. **e.** Skin thickness. **f.** Bulb diameter. **g.** HCS. h. HFs (%). Scale bar: 100 µm.

We investigated the effect of kartogenin on the anagen-to-catagen transition in mice HFs *in vivo*. Mice that underwent depilation to induce anagen phase, were treated with kartogenin (10 µM) or vehicle by subcutaneously injection from p.d.12 to p.d.17, and then their HF morphology at p.d.18 and p.d.21 were examined (Fig. 4a). The changes of mice dorsal skin in each group were analyzed at p.d.0, p.d.12, p.d.18. Our results indicated that the administration of kartogenin signifitantly prolonged the anagen phase compared with control group (Fig. 4b). Further we measured the length of refreshed hair from each group at p.d.18, and the kartogenin group showed longer hair than control mice at p.d.18 (p<0.05) (Fig 4c). Histomorphometric analysis of H&E -stained tissue sections revealed that kartogenin-treated HFs (Fig. 4d) were in anagen phase VI, with significantly larger bulb diameter and thicker skin compared with the control group (P<0.01) (Fig. 4d-f). We also calculated HCS and HF (%) for a more accurate determination of the hair growth cycle stage. The total HCS was lower in the kartogenin compared with the control group (P<0.01) (Fig. 4g), with 14.86% of HFs in the catagen IV-VI stage in the treated vs. approximately 36.13% in the control group (Fig. 4h).

**Figure 5:**
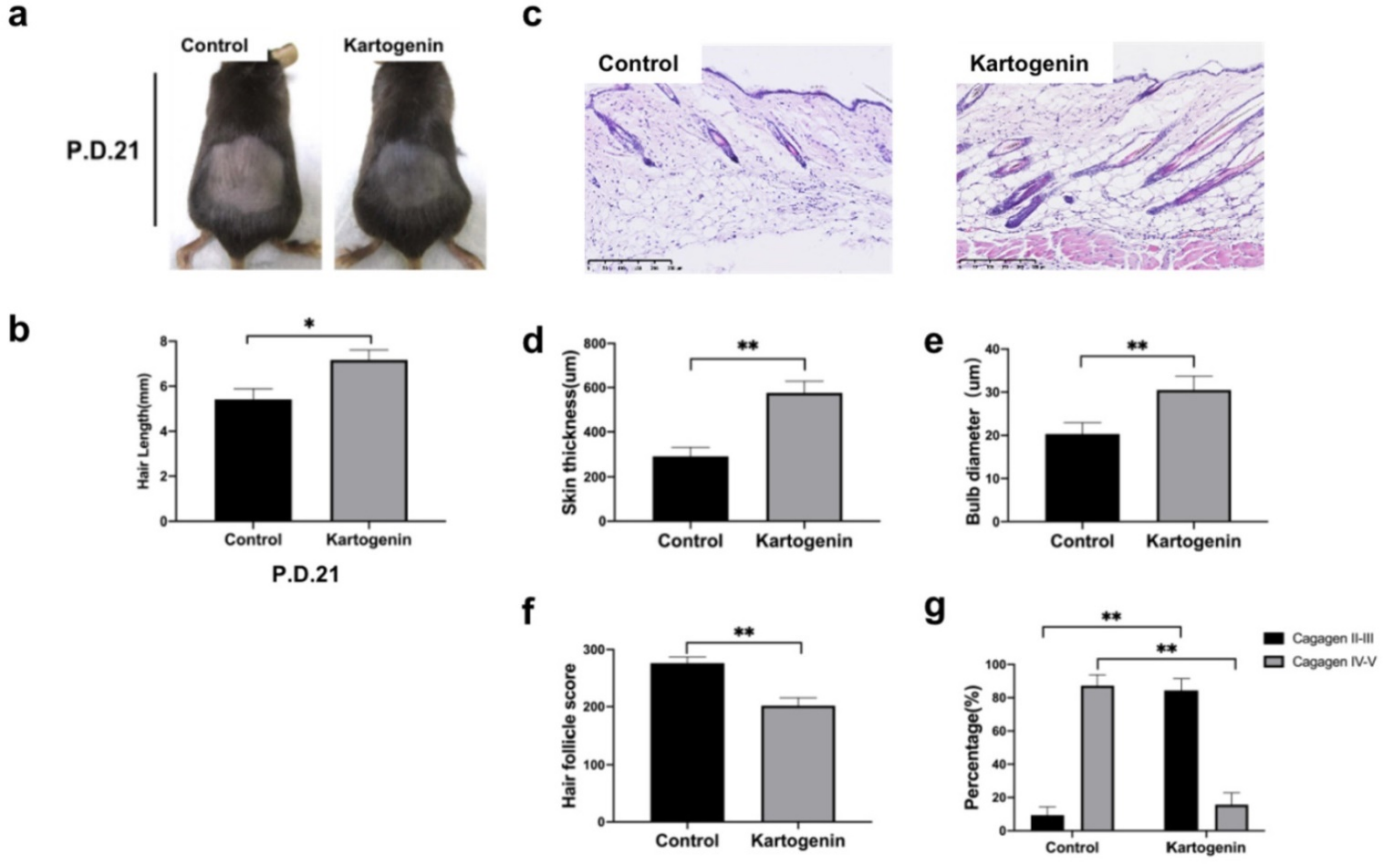
** Injection of kartogenin prolonged anagen phase and increased hair length. a.** Macrophotograph of control group and kartogenin group at p.d.21. **b.** Hair length. **c.** H&E staining images of control group and kartogenin group skin sample at p.d.21. **d.** Skin thickness. **e.** Bulb diameter. **f.** HCS. **g.** HFs (%). Scale bar: 100 µm. Results represent mean ± SD (n = 6 per group). *P < 0.05, **P < 0.01.

To better know the effects of kartogenin on hair growth, we further collected the dorsal skin sample at p.d.21 (Fig. 5), we found that at p.d.21, HFs of the control group were mainly in late catagen phase while HFs of kartogenin group just started to enter into early catagen phase (Fig. 5a, 5c-g). Moreover, we measured the length of new hairs of the depilated region at p.d.18 (Fig. 4c) and p.d.21 (Fig. 5c), we found out that injection of kartogenin resulted in longer hair shaft both at p.d.18 and p.d.21 by 1.20-fold and 1.32-fold higher respectively (p<0.05). These results indicated that kartogenin could delay anagen-to-catagen transition and promote hair regeneration.

## Discussion

The results of this study demonstrated that kartogenin regulated HF cell growth and the hair growth cycle transition. Kartogenin stimulated the proliferation and migration of cultured human ORSCs *in vitro*, which are necessary steps in HF growth and development. Furthermore, direct injection of kartogenin into murine HFs in anagen phase delayed catagen phase progression, accompanied by longer HF shafts, larger bulbs, and thicker skin. These effects were accompanied by downregulation of TGF-β2/Smad signaling pathway molecules.

To evaluate the efficacy of kartogenin *in vivo*, anagen phase was induced in C57BL/6 mice by depilation, according to a widely used model 29, and kartogenin was then injected subcutaneously at depilation-induced anagen phase time point (p.d. 12). Histological analysis showed that kartogenin delayed the anagen-to-catagen transition, thus prolonging the anagen phase and enlonged the length of hair shafts. Both men and women would experience hair loss 30,31. Burg et al. demonstrated a reduction in the proportion of HFs in anagen phase in patients with AGA and female pattern hair loss (FPHL) 30. Our current *in vitro* findings demonstrated that kartogenin promoted hair cell growth and migration in a dose-dependent manner. The behaviors of ORSCs are mainly influenced by rapid remodeling of epithelial keratinocytes 30 and are critical characteristics of the hair anagen-to-catagen transition, showing high proliferation and migration capacities during anagen phase and apoptotic features in catagen phase 32. These features suggested that kartogenin may be effective in hair loss by prolonging the anagen phase.

Injection of mice with kartogenin in the anagen phase delayed entry into catagen phase, similar to the effect on HFs in TGF-β-null mice, which increased hair cell proliferation and morphological suppression of catagen phase with longer anagen phase 33. Based on the effects of kartogenin on human ORSCs and the prolongation of anagen phase in mice, we hypothesized that TGF-β2 might be involved in kartogenin-induced anagen phase maintenance and hair growth cycle transitions 27.

To the best of our knowledge, the effects of kartogenin on TGF-β2 signaling have not been investigated previously. TGF-β2 signaling is a key signaling pathway in HF growth and development, through suppressing epithelial cell growth 19, 21, 34 and regulating apoptosis via p-Smad2 and p-Smad3 20. Furthermore, as a catagen phase-inducer, TGF-β2 inhibits hair growth and development by reducing anagen phase and accelerating the transition from anagen phase into catagen phase 19, 27, 34. Suppression of TGF-β2 and p-Smad2/3 activities thus prolongs the anagen phase and increases hair cell proliferation 34, 35. Inhibition of TGF-β2/Smad signaling is therefore a well-accepted method of treating most hair loss types, including alopecia induced by drugs, androgens, and psychological factors. The results of the present study revealed that kartogenin downregulated TGF-β2 mRNA and protein expression levels in cultured human ORSCs, and also downregulated other TGF-β2/Smad signaling pathway molecules in a dose-dependent manner.

Kartogenin possesses several advantages as a potential therapy for alopecia. It has shown excellent biocompatibility 12 and has demonstrated no toxicity in various cell types and no obvious adverse effects in animals 11, 12, 36. Furthermore, kartogenin is a highly stable, non-protein small molecule that can be cheaply and easily synthesized 36, and which can be easily stored and transported at room temperature 6, 12. In addition, similar to finasteride, which is the most widely used oral drug for androgenic alopecia, kartogenin could also suppress the anagen-to-catagen phase transition in HFs to prolong anagen phase 37, so a possible additive or synergy effect may exist between finasteride and kartogenin. Kartogenin is thus a promising new drug for the treatment of anagen-reduced hair loss. The results of this study provided novel evidence for the function of kartogenin in regulating HF growth and hair growth cycle transition.

## Supplementary Material

Supplementary figure.

## Figures and Tables

**Table 1:** Sequences of primers used for RT-PCR

Gene	Primer	Sequence
GAPDH	Forward	5′-CTCACCGGATGCACCAATGTT-3′
	Reverse	5′-CGCGTTGCTCACAATGTTCAT-3′
TGF-β2	Forward	5′-CATCCCGCCCACTTTCTAC-3′
	Reverse	5′-AATCCGTTGTTCAGGCACTC-3′
Smad2	Forward	5′-CCGACACACCGAGATCCTAAC-3′
	Reverse	5′-GAGGTGGCGTTTCTGGAATATAA-3′
Smad3	Forward	5′-CCATCTCCTACTACGAGCTGAA-3′'
	Reverse	5′-CACTGCTGCATTCCTGTTGAC-3′
