# Supplementary material for: Kartogenin regulates hair growth and hair cycling transition
Source: Int J Med Sci. 2022 Mar 6;19(3):537–45. doi: 10.7150/ijms.68434 (PMC8964329; doi:10.7150/ijms.68434)

Supplementary materials

Supplementary Fig.1

a-c. Full gel images for statistics of panel d from figure 3.

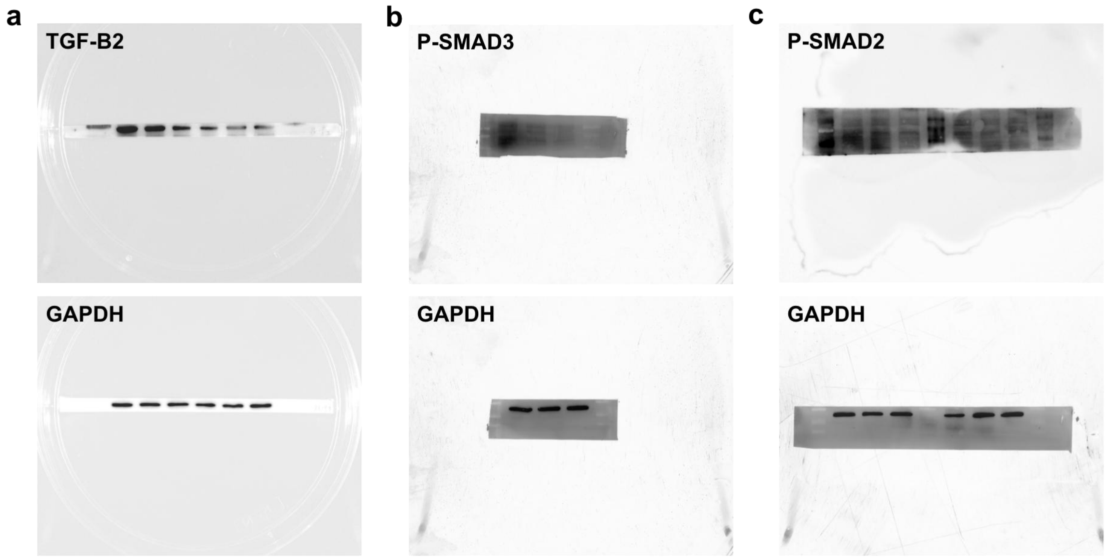

Supplement: Supplementary file 1 — Supplementary figure. [file ijmsv19p0537s1.pdf]
